# Locoregional Recurrence Prediction Using a Deep Neural Network of Radiological and Radiotherapy Images

**DOI:** 10.3390/jpm12020143

**Published:** 2022-01-21

**Authors:** Kyumin Han, Joonyoung Francis Joung, Minhi Han, Wonmo Sung, Young-nam Kang

**Affiliations:** 1 Department of Biomedicine & Health Sciences, College of Medicine, The Catholic University of Korea, Seoul 06591, Korea; rbals3216@gmail.com; 2 Advanced Institute for Radiation Fusion Medical Technology, College of Medicine, The Catholic University of Korea, Seoul 06591, Korea; 3 Department of Chemistry and Research, Institute for Natural Science, Korea University, Seoul 02841, Korea; joonyoungsun12@gmail.com (J.F.J.); minhihan224@gmail.com (M.H.); 4 Department of Biomedical Engineering, College of Medicine, The Catholic University of Korea, Seoul 06591, Korea; 5 Department of Radiation Oncology, Seoul St. Mary’s Hospital, College of Medicine, The Catholic University of Korea, Seoul 06591, Korea

**Keywords:** head and neck squamous cell carcinoma, deep learning, locoregional recurrence

## Abstract

Radiation therapy (RT) is an important and potentially curative modality for head and neck squamous cell carcinoma (HNSCC). Locoregional recurrence (LR) of HNSCC after RT is ranging from 15% to 50% depending on the primary site and stage. In addition, the 5-year survival rate of patients with LR is low. To classify high-risk patients who might develop LR, a deep learning model for predicting LR needs to be established. In this work, 157 patients with HNSCC who underwent RT were analyzed. Based on the National Cancer Institute’s multi-institutional TCIA data set containing FDG-PET/CT/dose, a 3D deep learning model was proposed to predict LR without time-consuming segmentation or feature extraction. Our model achieved an averaged area under the curve (AUC) of 0.856. Adding clinical factors into the model improved the AUC to an average of 0.892 with the highest AUC of up to 0.974. The 3D deep learning model could perform individualized risk quantification of LR in patients with HNSCC without time-consuming tumor segmentation.

## 1. Introduction

More than 650,000 cases of head and neck cancer including head and neck squamous cell carcinoma (HNSCC) have been reported worldwide annually, making it the seventh most common cancer [1,2]. To treat HNSCC, radiation therapy is frequently considered [3]. The locoregional recurrence (LR) varies depending on the primary site and stage and ranging from 15% to 50% after treatment of HNSCC [4,5,6,7,8]. In addition, the outcome of patients with LR after treatment is poor, with a 5-year survival rate of 50% [4,5,6]. To find high-risk patients who might suffer from LR in advance, it is necessary to develop a model capable of predicting the LR.

To predict the LR, radiomics has been widely used and proved to be powerful for predicting the prognosis of tumor after it is treated [8,9,10,11,12,13,14,15,16,17]. In addition, the prediction performance can be further improved using combined radiomics and dosiomics [16]. Although tumor segmentation and feature extraction are essential parts of -omics prediction, they are not integrated into a single model. Consequently, such -omics methods are hardly reproducible in other institutions [17,18,19,20]. Therefore, it is necessary to deal with radiological and radiotherapy images themselves, instead of radiomics and dosiomics.

A deep neural network (DNN) has been attracting attention as an innovative method for image analysis, natural language processing, and various research methods [12,21,22,23,24,25], because it can learn the rules governing the underlying phenomena directly from the data. Therefore, a solution for such problems can be solved by using a deep neural network. Combining DNNs with radiomics shows great potential to predict cancer patient prognosis following treatment [15,26]. One advantage of DNNs is that a DNN does not need a process of segmentation or feature extraction because DNN can capture features from images by training. Moreover, a DNN has the ability to extract features that cannot be described by traditional analytic algorithms.

To predict the prognosis of HNSCC, several methods based on radiomics have been reported [14,15]. Such methods based on radiomics have demonstrated that LR, distant metastasis, and overall survival of HNSCC can be predicted. In these studies, predicting models were evaluated using area under the curve (AUC) of receiver operating characteristic (ROC) curves. Diamant et al. have reported that they could achieve an AUC of 0.92 for predicting distant metastasis [15]. However, the AUC for predicting LR has been to be 0.65 by Diamant et al. and 0.69 by Vallières et al. [14,15], indicating that predicting distant metastasis and overall survival is more reliable than predicting LR. As AUC for predicting LR was found to be relatively low, the performance of predicting LR remains an issue.

In this work, we proposed a convolutional neural network (CNN) to predict the LR of patients with HNSCC following radiation therapy [1,3,12,27,28,29,30]. CNN-based deep learning models were designed to be capable of predicting LR using CT, FDG-PET, dose distribution, or clinical factors. In our model, medical images and does distributions were not segmented. Their features were not extracted to avoid the issue related to reproducibility [17,18,19,20]. In addition, five different models with different input configurations were compared. We found that the deep learning model receiving three images and additional clinical factors as input could predict the LR most effectively. This will be discussed below in great detail.

## 2. Materials and Methods

Data set: We used the data set containing FDG-PET/CT and radiotherapy planning CT imaging data of 298 patients with HNSCC in The Cancer Imaging Archive (TCIA) [14]. After excluding incomplete data set, data of 157 patients were used for this study as summarized in Table S1. For cross-validation, the data set was randomly divided into a training set (134 patients) and a test set (23 patients) for 5 times as summarized in Table S1. *p*-values were calculated by Fisher’s exact test to compare training and validation data sets.

Image preprocessing: CT and PET images and radiation dose distribution were cropped and interpolated into a total size of 300 mm × 300 mm × 99 mm with a voxel size of 3 mm × 3 mm × 3 mm. Homemade MATLAB^®^ (The MathWorks Inc., Natick, MA, USA) codes were used to crop and interpolate them.

**Figure 1:**
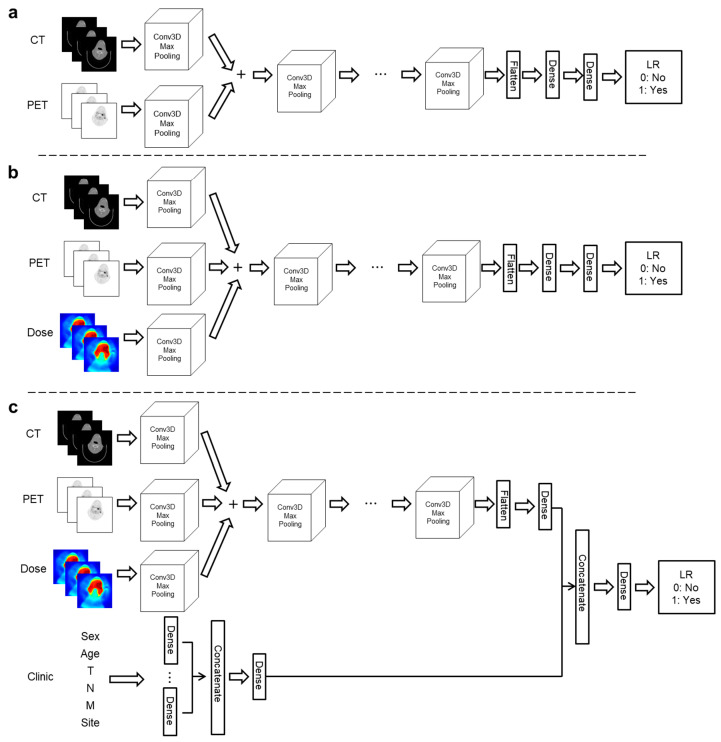
Deep learning model for predicting locoregional recurrence (LR): (**a**) DL model for CP (CT+PET) model. (**b**) CPD (CT+PET+Dose) model. (**c**) CPD-C (CT+PET+Dose+clinical factors) model. Architectures of DL models in detail can be found in Supporting Information.

Deep learning model: We developed a three-dimensional deep learning architecture as shown in Figure 1. We proposed three types of 3D DL architectures depending on the number of inputs (two, three, and four major inputs). Two inputs architectures were CP (CT+PET), CD (CT+Dose), and PD (PET+Dose) models (Figure 1a and Table S2). We also considered architectures (CPD model: CT+PET+Dose) with three inputs (Figure 1b). Finally, the CPD-C (CT+PET+Dose+clinical factors) architectures in Figure 1c included baseline patient characteristics such as sex, age, tumor stages, and primary disease site.

As CT and PET images with dose distribution were three dimensional (3D) images, 3D convolutional neural network (3D CNN) was used. For each model, conv3D and max pooling layers had window sizes of 3 × 3 × 2 and 2 × 2 × 2, respectively. After passing the first con3D and max pooling layers, hidden layers were summed to integrate information. To perform deep extraction of various features from inputs, channels of conv3D were set to be 16, 32, 64, 128, and 256. After setting all con3D and max pooling layers, vectors were flattened and passed through the one multi-layer perceptron to predict LR. In the CPD-C model, additional inputs of clinical factors were vectorized using the one-hot encoding method. Those inputs were integrated as shown in Figure 1c.

A rectified linear unit (ReLU) was used for activation functions of all layers except the last layer to predict LR. The activation function of the last layer was set to have a sigmoid function. Total parameters of models in Figure 1a–c were about 911, 911, and 915 thousand, respectively.

To train 3D DL models, images of a total of 157 patients were randomly divided into a training set (134 patients) and a test set (23 patients). As the number of images was relatively small, images were flipped in left–right, anterior–posterior, and inferior–superior directions. All combinations of flipping directions were applied to produce 8 times larger training data sets. Such data augmentation is a useful strategy to deal with small data set [31]. Three-dimensional DL models were optimized until the minimum validation loss was achieved as shown in Figure S1. Five randomly divided sets were used to train and test 3D DL models. No significant difference (*p*-value > 0.10) was found between the training and test sets for each data set by calculating Fisher’s exact test. Our 3D DL models were built using the Keras and trained using a computer with an intel CPU (i7-6700) and 16 GB of RAM.

## 3. Results

### 3.1. Features Captured by 3D DL Models

**Figure 2:**
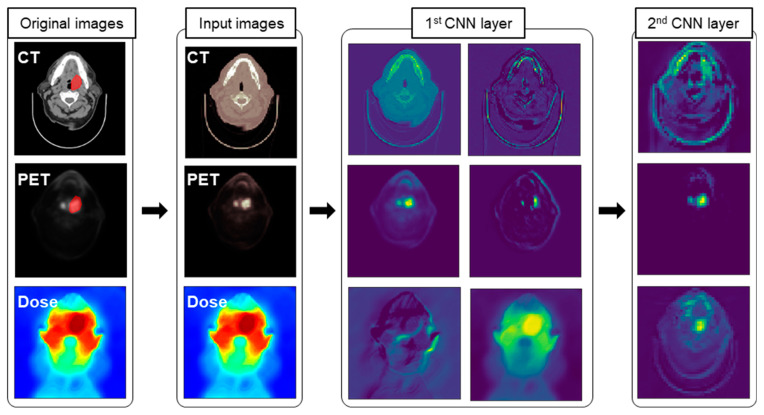
Visualized activation maps of the CPD-C (CT+PET+Dose+clinical factors) model. The first, second, and third rows of activation maps in the 1st CNN layer were extracted from conv3d_1, conv3d_2, and conv3d_3, respectively (See the architecture in Table S3). Activation maps in the 2nd CNN layer were extracted from conv3d_4.

In the case of DL model using CNN, each channel in 3D convolutional (conv3D) layers could capture features in images. Figure 2 depicts several selected activation maps captured by conv3D layers in the CPD-C model. A close inspection of feature map revealed the area around the tumor (highlighted). In the first row of the first CNN layer, activation maps resulting from CT images as input could capture overall shape and boundary. However, the area around the tumor was highlighted in activation maps by the layer that passed PET images. Activation maps produced by dose distributions captured a relatively complex pattern in the dose distribution. Therefore, our 3D DL models were found to have the ability to capture tumor related features to predict LR. As shown in Figures S2–S6, activation maps tuned into a more abstract representation to relate LR.

### 3.2. LR Prediction Using 3D DL Models

**Figure 3:**
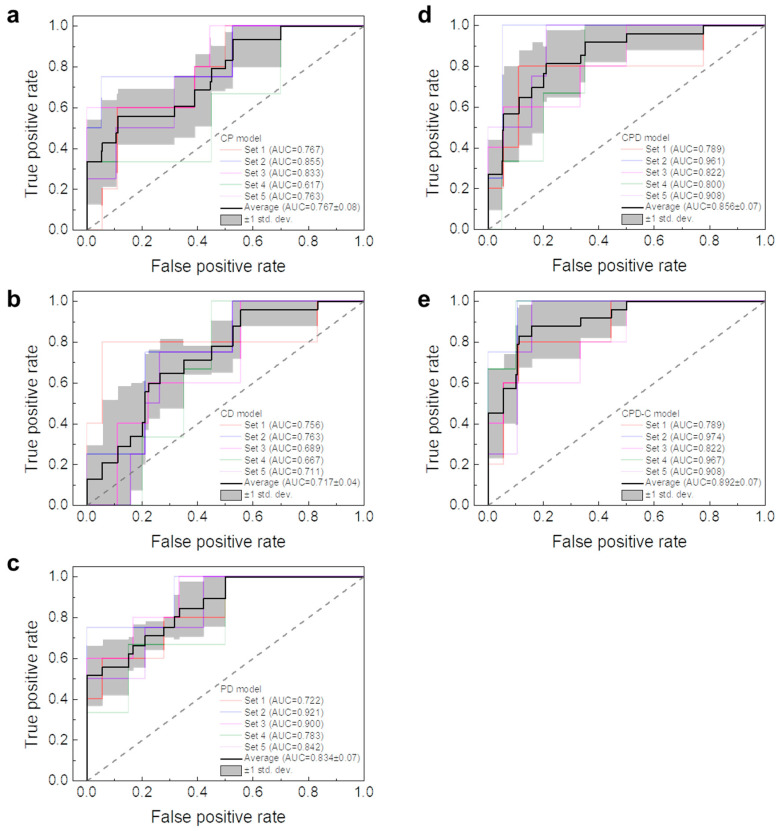
ROC curves of: (**a**) CP (CT+PET), (**b**) CD (CT+Dose), (**c**) PD (PET+Dose), (**d**) CPD (CT+PET+Dose), and (**e**) CPD-C (CT+PET+Dose+clinical factors) models.

The most accurate 3D DL model was found to be the CPD-C model with an average accuracy of 88.7% ± 4%. In addition, our 3D DL model required only 1.2 s to predict the LR after training using a computer with an intel CPU (i7-6700) and 16 GB of RAM. Three-dimensional DL models were further evaluated using area under the curve (AUC) of receiver operating characteristic (ROC) curves. The ROC curve is a graph of the false positive rate and the true positive rate obtained by changing the threshold value, which is the criterion for distinguishing positive and negative. Since our DL model was a binary classifier that distinguished whether or not LR occurred, the AUC of the ROC curve was used to compare five different 3D DL models. Results are shown in Figure 3. The CPD-C model was found to have the largest average AUC of 0.892 ± 0.07. AUCs were gradually increased in the order of CD (0.72 ± 0.04), CP (0.77 ± 0.07), PD (0.83 ± 0.07), and CPD (0.86 ± 0.07) models.

## 4. Discussion

Several studies have predicted the LR of HNSCC patients using various radiomics methods as summarized in Table 1. After feature extraction from CT and PET images, a method of finding the correlation with LR using a multivariate Cox proportional hazard regression model has been reported [16]. In this method, concordance index (CI) values were found to be 0.60, 0.66, and 0.56 for CP, CPD, and CPD-C models, respectively. Meanwhile, Wang et al. have reported a machine learning model such as a support vector machine using extracted features to predict LR [8]. In their study, AUCs of CP and CP-C were 0.76 and 0.77, respectively. In the group reporting the HNSCC data set to TCIA, AUC values of 0.64 and 0.69 were reported when LR was predicted with a random forest model using CP and CP-C [14]. Methods attempted to predict LR so far have been based on feature extraction. Recently, Diamant et al. have reported a CNN model for predicting the LR from central tumor slice, with an AUC of 0.65 [15].

Radiomic features such as first-order statistics and shape features are extracted after segmentation [16]. However, there is a risk of being missed if there is an unexpected effect between divided areas. In addition, unexpected image distortion may in discretizing CT and PET image with continuous values for feature extraction. Furthermore, features may have relatively shallow information because feature values are calculated according to a predetermined algorithm. However, features extracted by 3D DL model can be considered as more complex features than traditional features. Moreover, features are mixed to produce more abstract and informational features by passing through conv3D layers. After passing through the last con3D layer, features are transformed into the most abstract representation as shown in Figure S6.

In this study, the use of a 3D DL model was able to achieve a higher AUC than the conventional method. In the case of CP model, the averaged AUC value was 0.77 ± 0.07, which was higher than that of 0.76 of Wang et al. and 0.64 of M. Vallières et al. [8,14]. Such a high AUC value was achieved by skipping segmentation process and replacing feature extraction with deep learning. Since segmentation might suffer from inter- and intra-observer variation, it is a huge advance to avoid potentially erroneous procedures and human intervention [17,18,19,20]. Furthermore, an additional benefit of skipping the segmentation process is that the time needed for prediction can be dramatically reduced to about a second. Considering the time for segmentation, our model takes a negligible amount of time to make a prediction.

The AUC of the CPD model was larger than that of the CP model. This means that dose distribution with information about the treatment plan plays an important role in predicting recurrence after radiation therapy. As reported by Song and coworkers, dose distribution plays a very important role in predicting LR [16]. As shown by Song and coworkers, not only radiomics based on CT and PET images, but also dosiomics using the same strategy for dose distribution in predicting LR are important.

The average AUC value of the CD model shown in Table 1 was found to be lower than those of CP and PD models. As PET images clearly indicate the metabolic characterization of tumoral microenvironments, PET images may play a substantial role in predicting LR [12,32,33]. As averaged AUC values were increased in the order of CP, CPD, and CPD-C, LR prediction was improved when the more information was given. In the case of the CPD-C model to which the clinical factor was added, the averaged AUC value was 0.892 ± 0.07, which was the highest among all other models. In addition, the highest AUC of 0.974 was achieved for the CPD-C model. When compared to the concordance index (CI) value of CPD-C model of Song and coworkers (CI = 0.56) [16], the averaged AUC value of our CPD-C model is much higher. This is because our 3D DL model can effectively handle the clinical factors by representing them into more appropriate features in dense_1 to dense_6 layers and learning the correlations between the clinical factors and images in dense_7 and dense_9 layers as summarized in Table S4. Considering that the AUC of the CPD model was 0.856 ± 0.07, the improvement of AUC by clinical factors indicates that important information for prediction is not present in medical images or dose distribution. Additionally, the averaged AUC values of CPD-C model for each primary site and T stage are investigated as summarized in Table S5. The averaged AUC values of oropharynx and T2 stage were found to be highest among primary sites and T stages, respectively. This is because our 3D DL model was able to learn effectively based on the largest number of datapoints of oropharynx (*n* = 109) and T2 stage (*n* = 57).

**Figure 4:**
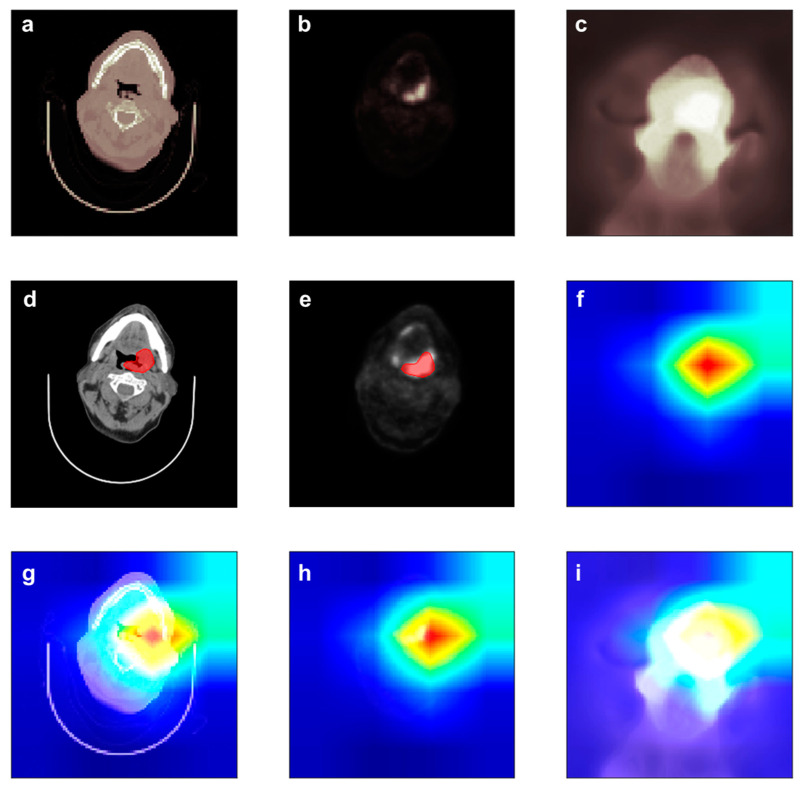
Heat map of LR positive patient produced by the CPD-C model: (**a**) Input images of CT. (**b**). Input images of PET. (**c**). Input images of dose distribution. (**d**). Contour images of CT. (**e**). Contour images of PET. (**f**). The heatmap. The red region in the heatmap indicates where the important region to make decision of LR. (**g**–**i**) are superimposed images of CT, PET, and dose distribution and heatmap, respectively.

DL models are often criticized in that it is hard to understand reason for the prediction the DL model made. This critique is called the Clever Hans effect, in which the DL model generates a correct answer for the wrong reason [34]. However, in the case of CNNs, the DL model shows an important part in making a prediction. Figure 4 shows the importance at the max_pooling3d_6 layer of the CPD-C model using Grad-CAM [35]. Importance values can be assigned by using the gradient information flowing into the last convolutional layer of the CNN in Grad-CAM [35].

Figure 4 depicts the contour and heatmap of an LR positive patient. The CPD-C model successfully predicted that the patient would be LR positive from images shown in Figure 4a–c. The red region in Figure 4f indicates where the important region captured by CPD-C model to predict LR. As shown in Figure 4g,h, the CPD-C model could recognize the area including the tumor area. In addition, it could be seen that a larger area including the tumor area was marked as important to predict LR. This indicates that not only the gross tumor volume (GTV), but also surrounding tissues are important for making a decision.

**Figure 5:**
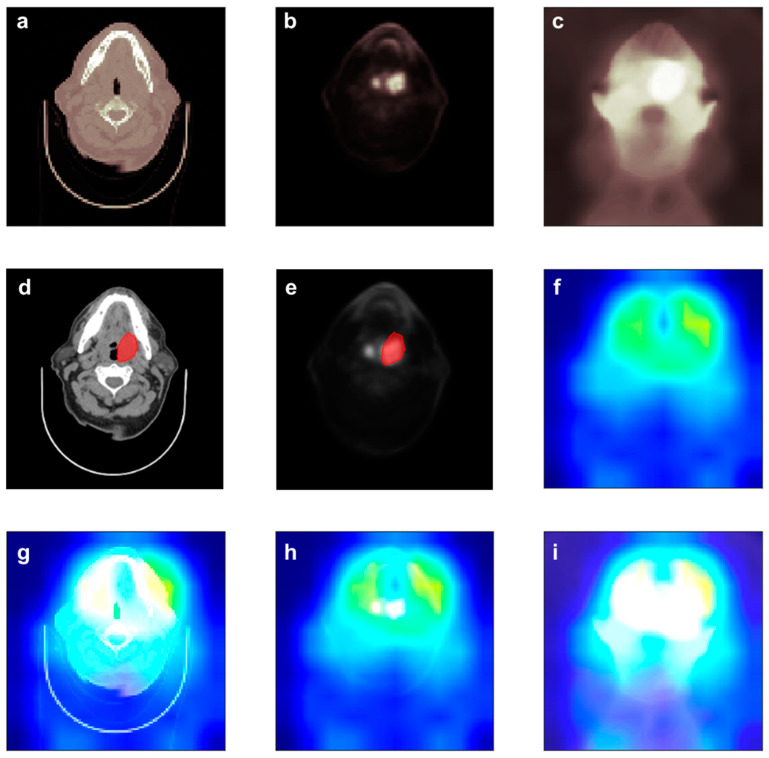
Heat map of an LR negative patient produced by the CPD model: (**a**). Input images of CT. (**b**). Input images of PET. (**c**). Input images of dose distribution. (**d**). Contour images of CT. (**e**). Contour images of PET. (**f**). The heatmap. The red region in the heatmap indicating the important region to make decision of LR. (**g**–**i**) are superimposed images of CT, PET, and dose distribution and heatmap, respectively.

In the case of the CPD model, the CPD model accurately predicted that the patient would be LR negative from images shown in Figure 5a–c. Similarly, the CPD model captured a larger area including the tumor area. Figures S7 and S8 depict the heat map of patients who are LR positive and negative, respectively. This indicates that our 3D DL model can make a decision by referring to complex features on images. In conventional radiomics, feature extractions were performed in the GTV area. It might miss information for surrounding tissues of tumor. However, 3D DL models showed that our model could overcome such issues by considering images themselves without segmentation or extracting complex features directly from images.

In our model, only six clinical factors (sex, age, T stage, N stage, M stage, and primary site) were considered to predict LR. In fact, human papilloma virus (HPV) is known to be related to HNSCC [12,36,37]. However, the infection of HPV was not handled in our model because information about HPV was lacking in the data set. The status of HPV infection might be important to improve our 3D DL model. In addition, there could be other unknown clinical factors, such as effects of chemotherapy, tobacco consumption, and alcohol abuse, related to LR of HNSCC. Deep learning method is useful to identify which clinical factors are important to improve model performance. Systematic research on finding critical clinical factors can open the opportunity to find high risk patients.

## 5. Conclusions

This study demonstrated the impact of deep learning on LR prediction through pretreatment images (CT/PET scans) and treatment schemes (dose distributions). The performance of LR prediction was increased when baseline patient characteristics were considered. Our model allowed prediction consistency and established a cost-effective system to meet large clinical demands with less manual intervention. The use of a non-invasive model with pretreatment information could predict patient prognosis, which can have a potential clinical implication on personalized therapy.

## Figures and Tables

**Table 1:** Averaged AUC of test set compared to AUCs of other previous studies.

	CP	CP-C	CD	PD	CPD	CPD-C
Song et al. [16]	0.60 ^a^	-	-	-	0.66 ^a^	0.56 ^a^
Wang et al. [8]	0.76	0.77	-	-	-	-
Vallières et al. [14]	0.64	0.69	-	-	-	-
Diamant et al. [15]	0.65	-	-	-	-	-
In this study	0.77 ± 0.07	-	0.72 ± 0.04	0.83 ± 0.07	0.86 ± 0.07	0.89 ± 0.07

^a^ Those values are concordance index (CI) values.

## Data Availability

The data set containing FDG-PET/CT and radiotherapy planning CT imaging data of 298 patients with HNSCC can be found at The Cancer Imaging Archive (TCIA) (https://doi.org/10.7937/K9/TCIA.2017.8oje5q00, accessed on 11 October 2021).

## Supplementary Materials

The following supporting information can be downloaded at: https://www.mdpi.com/article/10.3390/jpm12020143/s1, Figure S1: The training procedure of CPD-C model with set 1, Figures S2–S6: activation maps passing through the layers of CPD-C model in Table S3, and Figures S7 and S8: Heat map of CPD-C model for the images of patient, Table S1: The characteristics of 157 patients in head and neck squamous cell carcinoma data set., Tables S2–S4: The architecture of deep learning models, Table S5: Averaged AUC of CPD-C model for each primary site, T and N stage.
