# Supplementary material for: Locoregional Recurrence Prediction Using a Deep Neural Network of Radiological and Radiotherapy Images
Source: J Pers Med. 2022 Jan 21;12(2):143. doi: 10.3390/jpm12020143 (PMC8875706; doi:10.3390/jpm12020143)
Supplement: Supplementary file 1 [file jpm-12-00143-s001.zip › jpm-1438641-supplementary.pdf]

Table S1. The characteristics of 157 patients in head and neck squamous cell carcinoma dataset.

**Characteristics**	**Total *N* (%)**	Set 1 Train/Test	Set 2 Train/Test	Set 3 Train/Test	Set 4 Train/Test	Set 5 Train/Test
**Sex**						
M	121 (77.1)	103/19	105/17	106/16	103/19	107/15
F	33 (22.9)	31/4	29/6	28/7	31/4	27/8
p-value[1]		0.786	0.598	0.415	0.786	0.172
**T stage**						
T1	21 (13.4)	15/6	19/2	19/2	18/3	17/4
T2	57 (35.7)	49/8	47/10	50/7	50/7	48/9
T3	49 (31.8)	43/6	43/6	41/8	42/7	41/8
T4	22 (14.0)	21/1	19/3	21/2	17/5	21/2
Tx[2]	8 (5.1)	6/2	6/2	4/4	7/1	8/0
p-value[1]		0.186	0.754	0.101	0.839	0.743
**N stage**						
N0	19 (12.1)	18/1	18/1	17/2	17/2	18/1
N1	23 (14.0)	20/3	20/3	20/3	20/3	18/5
N2	103 (65.6)	88/15	84/19	88/15	86/17	87/16
N3	13 (8.3)	9/4	13/0	10/3	12/1	12/1
p-value[1]		0.275	0.242	0.783	0.888	0.564
**M stage**						
M0	155 (98.7)	133/22	132/23	133/22	132/23	133/22
Mx[3]	2 (1.3)	1/1	2/0	1/1	2/0	1/1
p-value[1]		0.272	1.000	0.272	1.000	0.272
**Primary site**						
Nasopharynx	14 (8.9)	11/3	13/1	12/2	11/3	11/3
Oropharynx	109 (69.4)	93/16	90/19	97/12	93/16	92/17
Hypopharynx	11 (7.0)	11/0	10/1	9/2	9/2	10/1
Larynx	16 (10.2)	14/2	15/1	12/4	15/1	14/2
unknown	7 (4.5)	5/2	6/1	4/3	6/1	7/0
p-value[1]		0.425	0.792	0.092	0.786	0.850
**Locoregional Recurrence**						
Yes	23 (15.3)	18/5	19/4	18/5	20/3	19/4
No	134 (84.7)	116/18	115/19	116/18	114/20	115/19
p-value[1]		0.337	0.749	0.337	1.000	0.749

[1] Comparisons between train and test datasets by Fisher's exact test.

[2] Tx: Main tumor cannot be measured.

[3] Mx: Metastasis cannot be measured.

Deep learning architectures

Deep learning models were made using the deep learning framework Keras. The architectures of CP (CT+PET), CD (CT+Dose), PD (PET+Dose), CPD (CT+PET+Dose), and CPD-C (CT+PET+Dose+clinical) models are summarized in Table S2, S3, and S4.

Table S2. The architecture of CP/CD/PD models

Layer (Type)<br>Filter size	Output shape	Param #	Connected to
input_1 (InputLayer)	100×100×33×1	0	
input_2 (InputLayer)	100×100×33×1	0	
conv3d_1 (Conv3D)<br>3×3×2×16	98×98×32×16	304	input_1
conv3d_2 (Conv3D)<br>3×3×2×16	98×98×32×16	304	input_2
max_pooling3d_1<br>(MaxPooling3D)<br>2×2×2	49×49×16×16	0	conv3d_1
max_pooling3d_2<br>(MaxPooling3D)<br>2×2×2	49×49×16×16	0	conv3d_2
add_1 (Add)	49×49×16×16	0	max_pooling3d_1<br>max_pooling3d_2
conv3d_3 (Conv3D)<br>3×3×1×32	47×47×16×32	4640	add_1
max_pooling3d_3<br>(MaxPooling3D)<br>2×2×1	23×23×16×32	0	conv3d_3
conv3d_4 (Conv3D)<br>3×3×2×64	21×21×15×64	36928	max_pooling3d_3
max_pooling3d_4<br>(MaxPooling3D)<br>2×2×2	10×10×7×64	0	conv3d_4
conv3d_5 (Conv3D)<br>3×3×2×128	8×8×6×128	147584	max_pooling3d_4
max_pooling3d_5<br>(MaxPooling3D)<br>2×2×2	4×4×3×128	0	conv3d_5
conv3d_6 (Conv3D)<br>3×3×2×256	2×2×2×256	590080	max_pooling3d_5
flatten_1 (Flatten)	2048	0	conv3d_6
dense_1 (Dense)	64	131136	flatten_1
dense_2 (Dense)	1	65	dense_1

Total Params: 911,041

Table S3. The architecture of CPD models

Layer (Type)<br>Filter size	Output shape	Param #	Connected to
input_1 (InputLayer)	100×100×33×1	0	(CT)
input_2 (InputLayer)	100×100×33×1	0	(PET)
input_3 (InputLayer)	100×100×33×1	0	(Dose)
conv3d_1 (Conv3D)<br>3×3×2×16	98×98×32×16	304	input_1
conv3d_2 (Conv3D)<br>3×3×2×16	98×98×32×16	304	input_2
conv3d_3 (Conv3D)<br>3×3×2×16	98×98×32×16	304	input_3
max_pooling3d_1<br>(MaxPooling3D)<br>2×2×2	49×49×16×16	0	conv3d_1
max_pooling3d_2<br>(MaxPooling3D)<br>2×2×2	49×49×16×16	0	conv3d_2
max_pooling3d_3<br>(MaxPooling3D)<br>2×2×2	49×49×16×16	0	conv3d_3
add_1 (Add)	49×49×16×16	0	max_pooling3d_1<br>max_pooling3d_2<br>max_pooling3d_3
conv3d_4 (Conv3D)<br>3×3×1×32	47×47×16×32	4640	add_1
max_pooling3d_4<br>(MaxPooling3D)<br>2×2×1	23×23×16×32	0	conv3d_4
conv3d_5 (Conv3D)<br>3×3×2×64	21×21×15×64	36928	max_pooling3d_4
max_pooling3d_5<br>(MaxPooling3D)<br>2×2×2	10×10×7×64	0	conv3d_5
conv3d_6 (Conv3D)<br>3×3×2×128	8×8×6×128	147584	max_pooling3d_5
max_pooling3d_6<br>(MaxPooling3D)<br>2×2×2	4×4×3×128	0	conv3d_6
conv3d_7 (Conv3D)<br>3×3×2×256	2×2×2×256	590080	max_pooling3d_6
flatten_1 (Flatten)	2048	0	conv3d_7
dense_1 (Dense)	64	131136	flatten_1
dense_2 (Dense)	1	65	dense_1

Total Params: 911,345

Table S4. The architecture of CPD-C model.

Layer (Type)<br>Filter size	Output shape	Param #	Connected to
input_1 (InputLayer)	100×100×33×1	0	(CT)
input_2 (InputLayer)	100×100×33×1	0	(PET)
input_3 (InputLayer)	100×100×33×1	0	(Dose)
conv3d_1 (Conv3D)<br>3×3×2×16	98×98×32×16	304	input_1
conv3d_2 (Conv3D)<br>3×3×2×16	98×98×32×16	304	input_2
conv3d_3 (Conv3D)<br>3×3×2×16	98×98×32×16	304	input_3
max_pooling3d_1<br>(MaxPooling3D)<br>2×2×2	49×49×16×16	0	conv3d_1
max_pooling3d_2<br>(MaxPooling3D)<br>2×2×2	49×49×16×16	0	conv3d_2
max_pooling3d_3<br>(MaxPooling3D)<br>2×2×2	49×49×16×16	0	conv3d_3
add_1 (Add)	49×49×16×16	0	max_pooling3d_1<br>max_pooling3d_2<br>max_pooling3d_3
conv3d_4 (Conv3D)<br>3×3×1×32	47×47×16×32	4640	add_1
max_pooling3d_4<br>(MaxPooling3D)<br>2×2×1	23×23×16×32	0	conv3d_4
conv3d_5 (Conv3D)<br>3×3×2×64	21×21×15×64	36928	max_pooling3d_4
max_pooling3d_5<br>(MaxPooling3D)<br>2×2×2	10×10×7×64	0	conv3d_5
conv3d_6 (Conv3D)<br>3×3×2×128	8×8×6×128	147584	max_pooling3d_5
max_pooling3d_6<br>(MaxPooling3D)<br>2×2×2	4×4×3×128	0	conv3d_6
conv3d_7 (Conv3D)<br>3×3×2×256	2×2×2×256	590080	max_pooling3d_6
flatten_1 (Flatten)	2048	0	conv3d_7
input_4 (InputLayer)	1	0	(Sex)
input_5 (InputLayer)	1	0	(Age)
input_6 (InputLayer)	8	0	(T stage)
input_7 (InputLayer)	9	0	(N stage)
input_8 (InputLayer)	3	0	(M stage)

input_9 (InputLayer)	6	0	(Primary disease site)
dense_1 (Dense)	3	6	input_4
dense_2 (Dense)	3	6	input_5
dense_3 (Dense)	3	27	input_6
dense_4 (Dense)	3	30	input_7
dense_5 (Dense)	3	12	input_8
dense_6 (Dense)	3	21	input_9
concatenate_1 (Concatenate)	18	0	dense_1<br>dense_2<br>dense_3<br>dense_4<br>dense_5<br>dense_6
dense_7 (Dense)	64	1216	concatenate_1
dense_8 (Dense)	64	131136	flatten_1
concatenate_2 (Concatenate)	128	0	dense_7<br>dense_8
dense_9 (Dense)	16	2064	concatenate_2
dense_10 (Dense)	1	17	dense_9

Total Params: 914,679

Table S5. Averaged AUC of CPD-C model for each primary site, T and N stage.

Site	Avg. AUC	T stage	Avg. AUC	N stage	Avg. AUC
Nasopharynx	0.92±0.09	T1	0.87±0.17	N0	0.80±0.15
Oropharynx	0.96±0.04	T2	0.97±0.03	N1	0.94±0.05
Hypopharynx	0.85±0.16	T3	0.88±0.13	N2	0.96±0.05
Larynx	0.85±0.17	T4	0.96±0.05	N3	0.93±0.10
unknown	0.93±0.08	Tx	0.94±0.07		

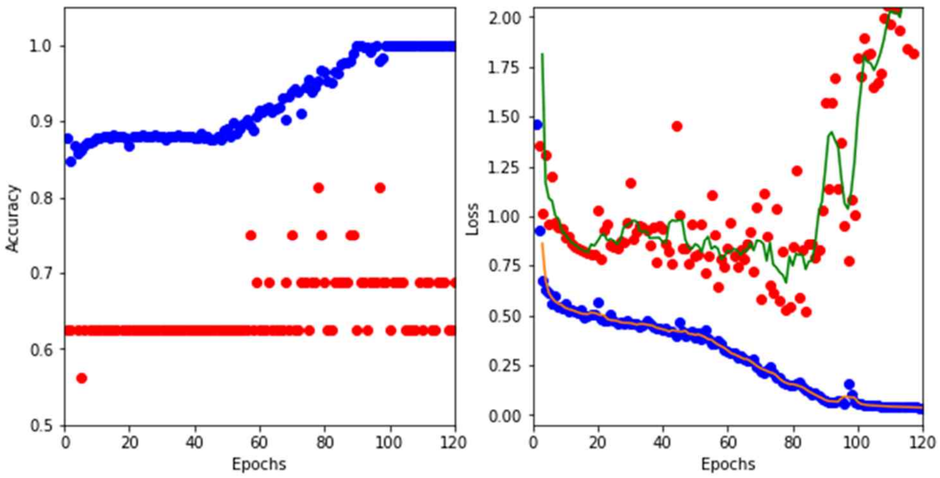

Figure S1. The training procedure of CPD-C model with set 1. a. The plot of epochs vs accuracy. **b**. The plot of epochs vs loss. The green line is moving average.

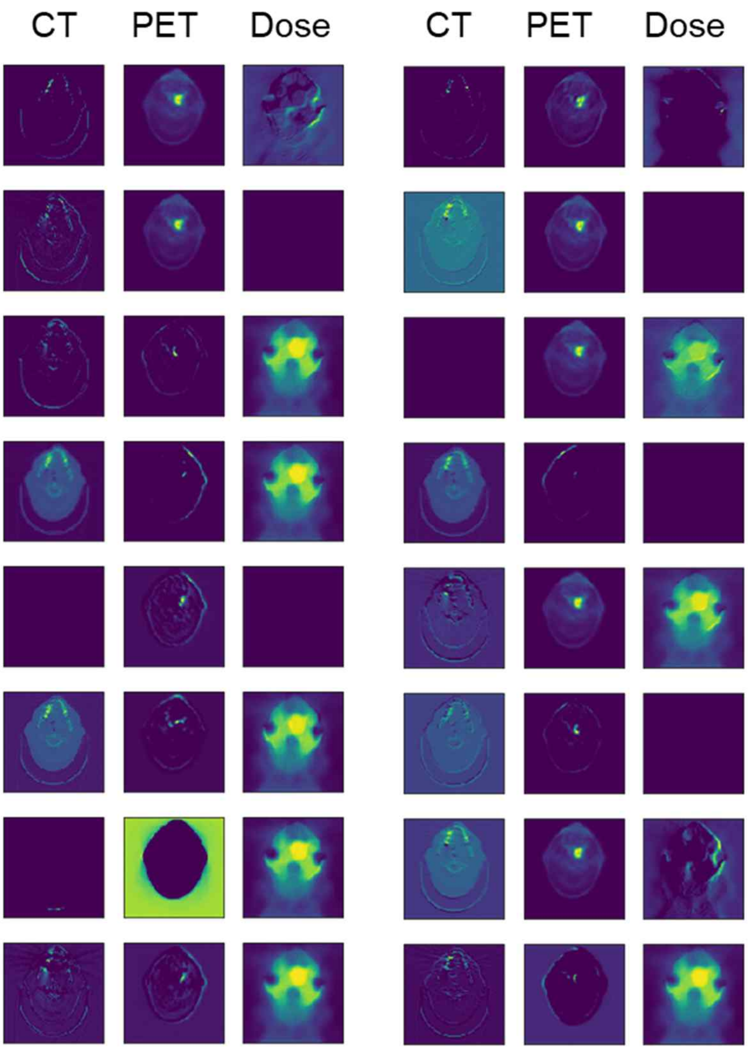

Figure S2. Total 16 activation maps passing through the conv3D_1, conv3D_2, and conv3D_3 layers of CPD-C model in Table S3.

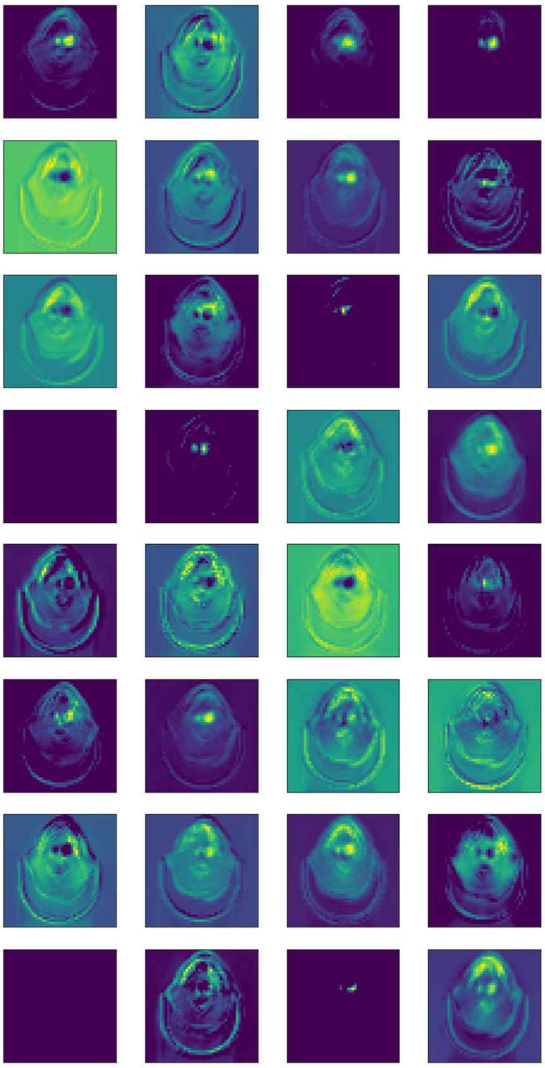

Figure S3. Total 32 activation maps passing through the conv3D_4 layer of CPD-C model in Table S3.

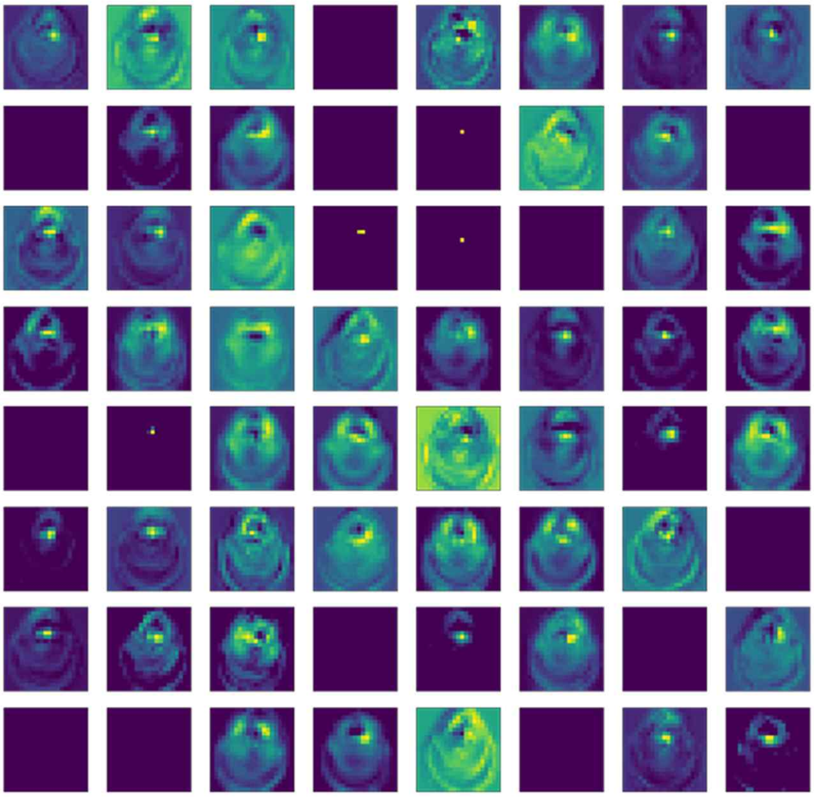

Figure S4. Total 64 activation maps passing through the conv3D_5 layer of CPD-C model in Table S3.

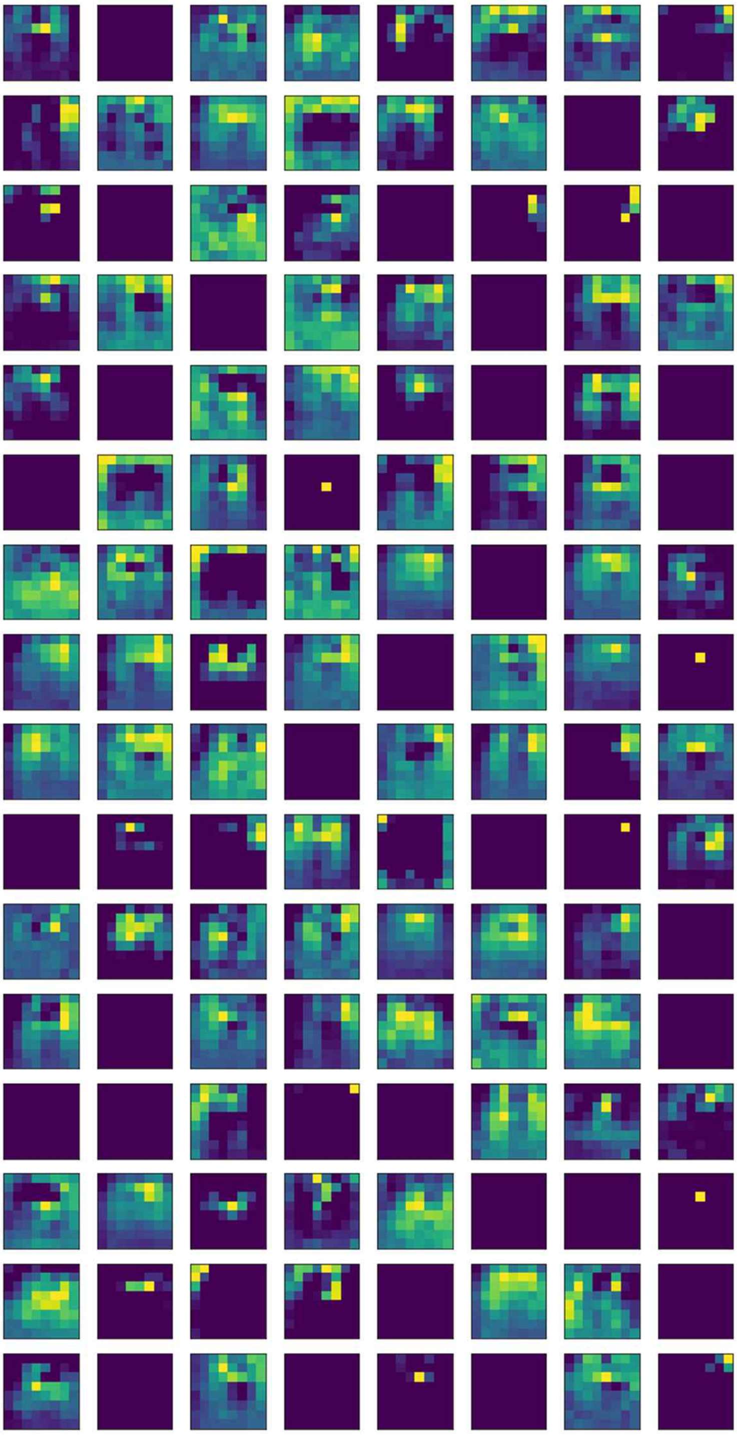

Figure S5. Total 128 activation maps passing through the conv3D_6 layer of CPD-C model in Table S3.

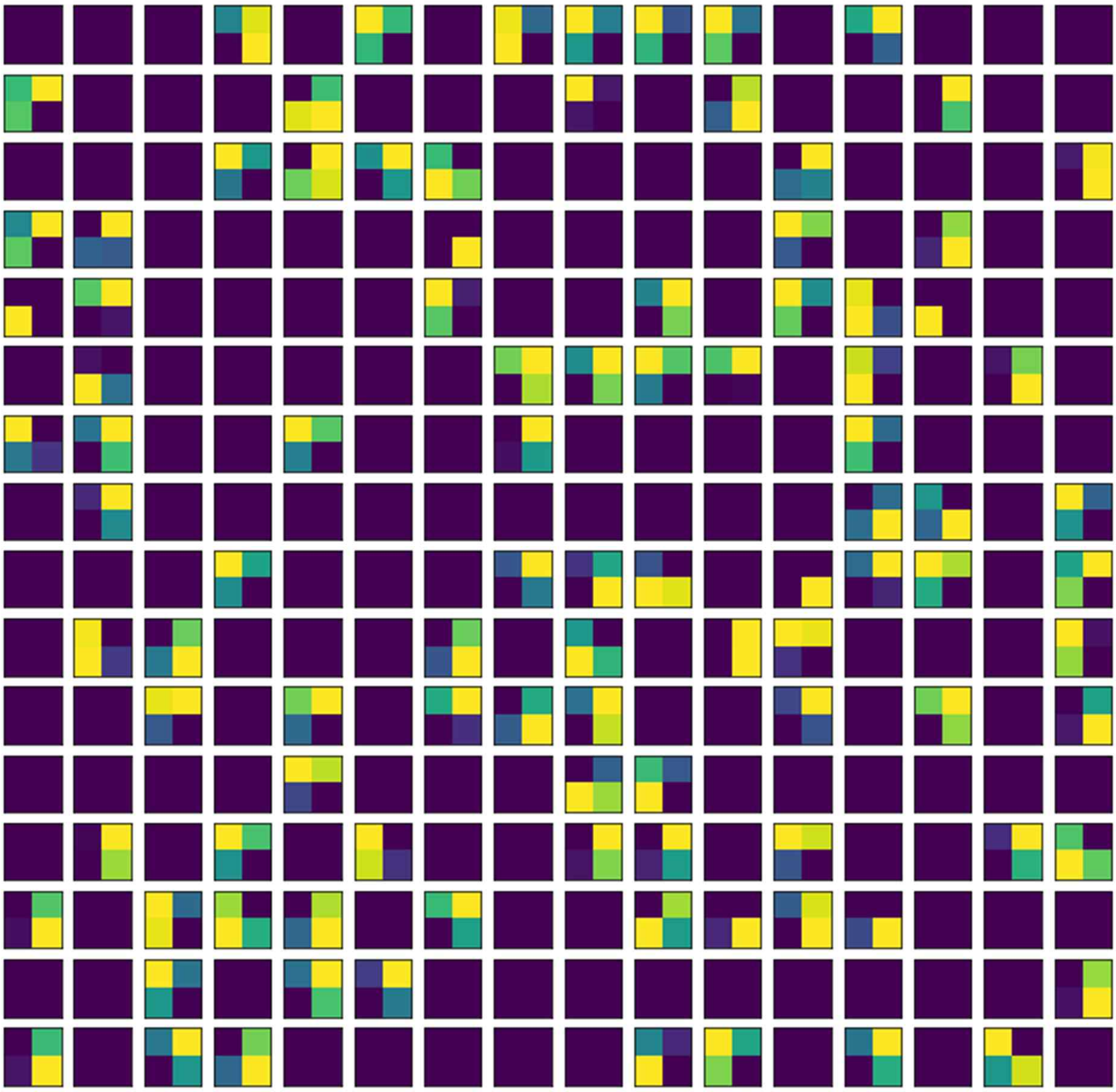

Figure S6. Total 256 activation maps passing through the conv3D_7 layer of CPD-C model in Table S3.

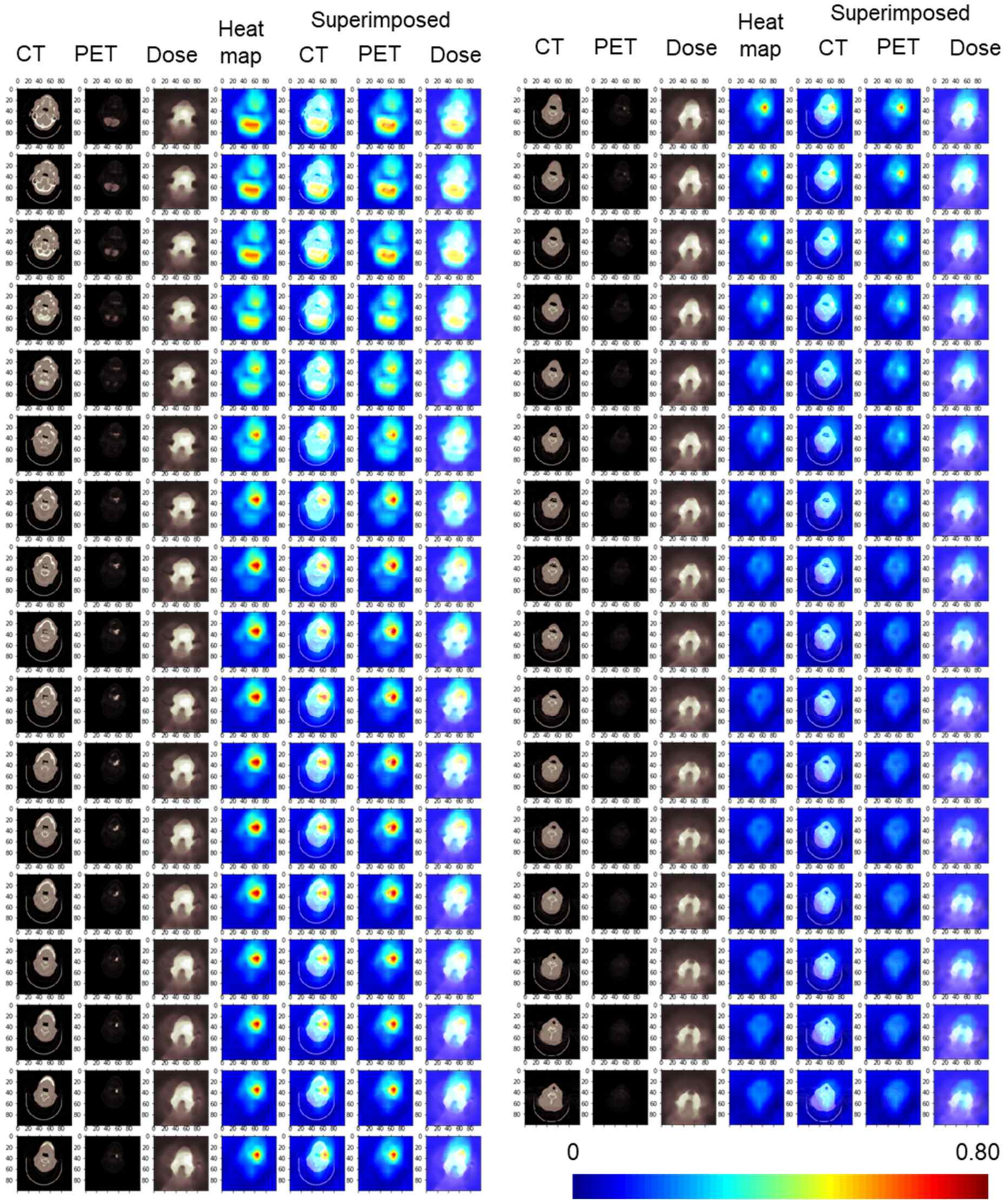

Figure S7. Heat map of CPD-C model for the images of patient who is LR positive.

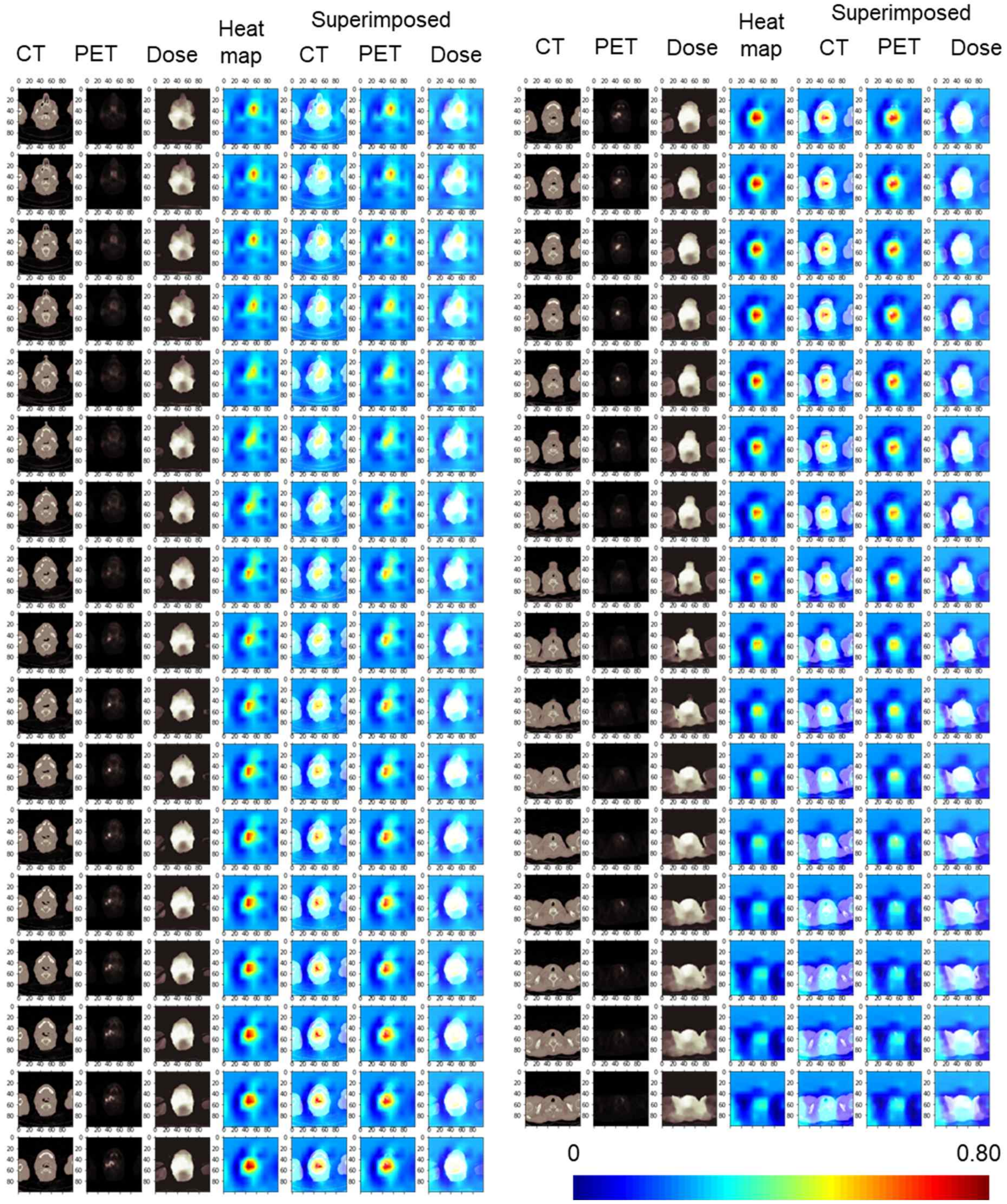

Figure S8. Heat map of CPD-C model for the images of patient who is LR negative.
